# Comparison of the Sagittal Spine Lordosis by Supine Computed Tomography and Upright Conventional Radiographs in Patients with Spinal Trauma

**DOI:** 10.1155/2014/967178

**Published:** 2014-05-21

**Authors:** Samy Bouaicha, Claudia Lamanna, Thorsten Jentzsch, Hans-Peter Simmen, Clément M. L. Werner

**Affiliations:** Division of Traumatology, University Hospital of Zurich, University of Zurich, Raemistraß 100, 8091 Zurich, Switzerland

## Abstract

*Study Design.* Retrospective data analysis. *Objective.* To compare the sagittal lordosis of the lumbar spine by supine computed tomography (CT) and upright conventional radiographs. *Summary of Background Data.* There is sparse data about position and modality dependent changes of radiographic measurements in the sagittal lumbar spine. *Methods.* The anatomical and functional Cobb angles of the thoracolumbar spine in 153 patients with spinal injury were measured by conventional upright sagittal radiographs and supine CT scans. Patients were assigned either to group A (*n* = 101), with radiologically confirmed vertebral fractures, or to group B (*n* = 52), without any osseous lesions. The interchangeability of the two imaging modalities was calculated using a ±3° and 5° range of acceptance. *Results.* Group A showed a mean intraindividual difference of −3.8° for both the anatomical and the functional Cobb angle. Only 25.7% and 27.7% of the 101 patients showed a difference within the tolerated ±3° margin. Using the ±5° limits, only 46 and 47 individuals fell within the acceptable range, respectively. In the patients in group B, the mean intraindividual difference was −2.1° for the anatomical and −1.5° for the functional Cobb angle. Of the 52 patients, only 14 and 13 patients, respectively demonstrated an intraindividual difference within ±3°. With regard to a threshold of ±5°, both the functional and anatomical values were within the defined margins in only 25 (48%) patients. *Conclusion.* The use of supine CT measurements as a baseline assessment of the sagittal lordosis of the injured thoracolumbar spine does not appear to be appropriate when upright conventional sagittal plane radiographs are used for follow-up measurements.

## 1. Introduction

Whole body computed tomography (WBCT) in the multiply injured patient is a valuable tool that can be used to assess the severity of trauma and determine prioritization in the course of treatment [1]. Using this tool, vertebral lesions are detected and routinely classified. Depending on the fracture type, concomitant lesions, and individual patient characteristics, some vertebral fractures are deemed appropriate for conservative treatment. These patients undergo clinical and radiological followup at regular intervals to reassess the geometry of the fractured vertebra(e) and the lordosis of the spine. Sagittal conventional radiographs in the upright position are usually performed, and the measurement of vertebral body height and Cobb angles is used to compare images.

Sagittal spine geometry is position-dependent, and therefore radiological measurements cannot be equated automatically from one position to another [2–5]. Furthermore, the interchangeability of radiological modalities is a commonly neglected issue in daily clinical practice that may also significantly alter the accuracy of pathological measurements [3, 6]. Only a few studies have examined position-dependent measurements and the interchangeability of radiological modalities in lumbar spine imaging [3]. No data are available regarding the interchangeability of supine CT measurements of the lumbar spine with upright conventional sagittal radiographs. Such interchangeability would be beneficial in all trauma cases for which index imaging is performed using whole body CT to avoid additional upright baseline radiographs in consideration of radiation exposure and cost reduction.

To our knowledge, the present study is the first to examine the possible interchangeability of the sagittal spine lordosis obtained by supine CT scan and upright conventional radiographs.

## 2. Material and Methods

### 2.1. Patients

We retrospectively analyzed a consecutive series of multiply injured patients from our hospital imaging database who underwent WBCT in the emergency department for rapid trauma assessment and a conventional X-ray, usually within a month, between September 2006 and November 2010. Both conventional upright radiographs and supine CT scans of the thoracolumbar spine were available for 153 patients with a clinical suspicion of spinal injury, comprising 78 males and 75 females (mean age: 56). All included patients were divided in two groups. Group A comprised all patients with one or more vertebral fractures (*n* = 101), and group B patients demonstrated no osseous lesions by conventional radiographs or CT scans (*n* = 52). Division into two groups was performed because we opted to resemble the situation in clinical practice where follow-up visits with conventional X-rays are necessary for patients with fractures as well as patients without fractures but persisting pain. Vertebral fractures consisted of type A fractures according to Magerl et al. [7] which were amenable to conservative treatment [8]. All patients with primary operative treatment of the spine were excluded.

### 2.2. Measurements by Conventional Radiographs

**Figure 1:**
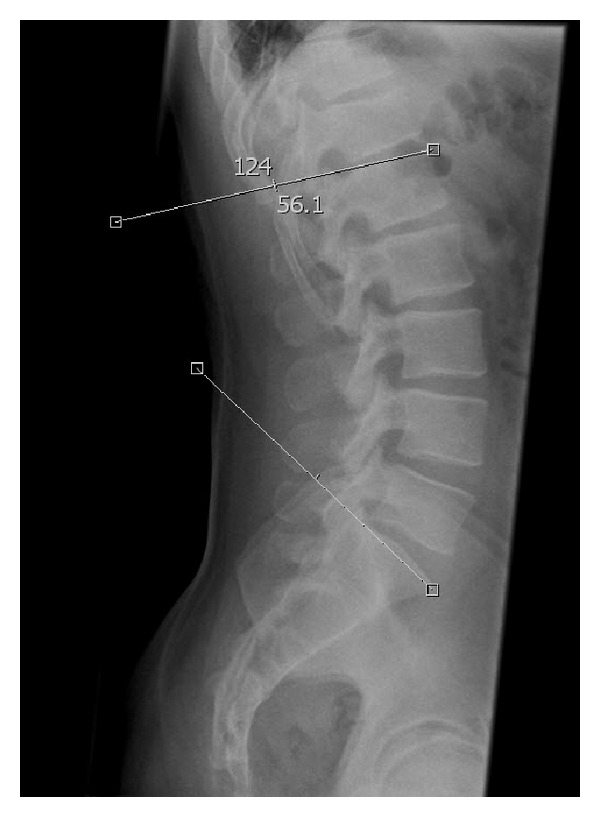
Anatomical Cobb angle measurement in conventional radiograph.

**Figure 2:**
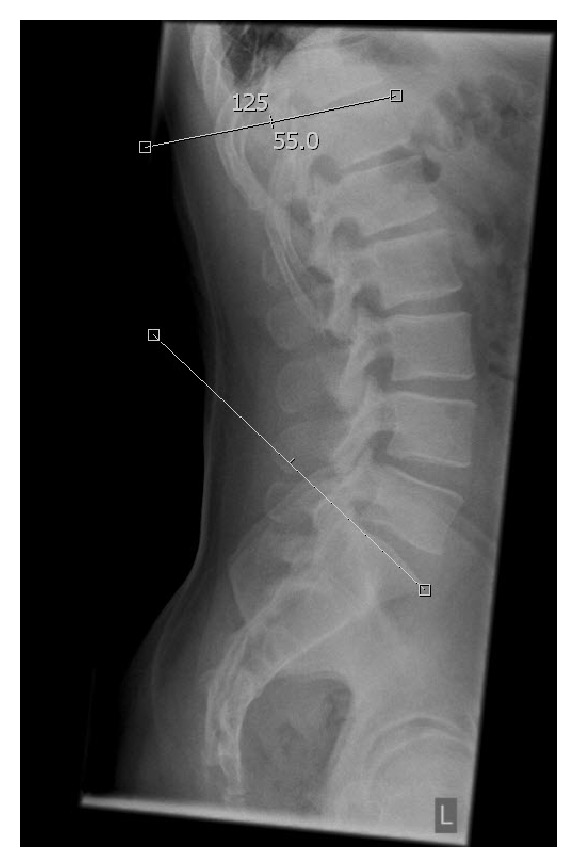
Functional Cobb angle measurement in conventional radiograph.

The sagittal lordosis of the lumbar and thoracolumbar spine was assessed by measuring the anatomical and functional Cobb angles on sagittal plain films in an upright position. While the anatomical Cobb angle of the lumbar spine was measured from the cranial end plate of S1 to the cranial end plate of L1 (Figure 1), the functional Cobb angle was measured according to Roussouly et al. [9], from the cranial end plate of S1 to the inflection point with respect to the cranial end plate of the neutral vertebra, whether it was localized cranial or caudal to L1 (Figure 2). Two independent measurements were performed for both the anatomical and functional Cobb angle in each case. First, tangents were aligned following the bodies of the end plate of S1 and the cranial end plate of L1. The second measurement was carried out with regard to other morphological characteristics of the vertebral bodies using a line drawn at the anterior and posterior corners of the vertebral bodies. All angles were measured digitally using a PACS system.

### 2.3. Measurements by CT Scans

**Figure 3:**
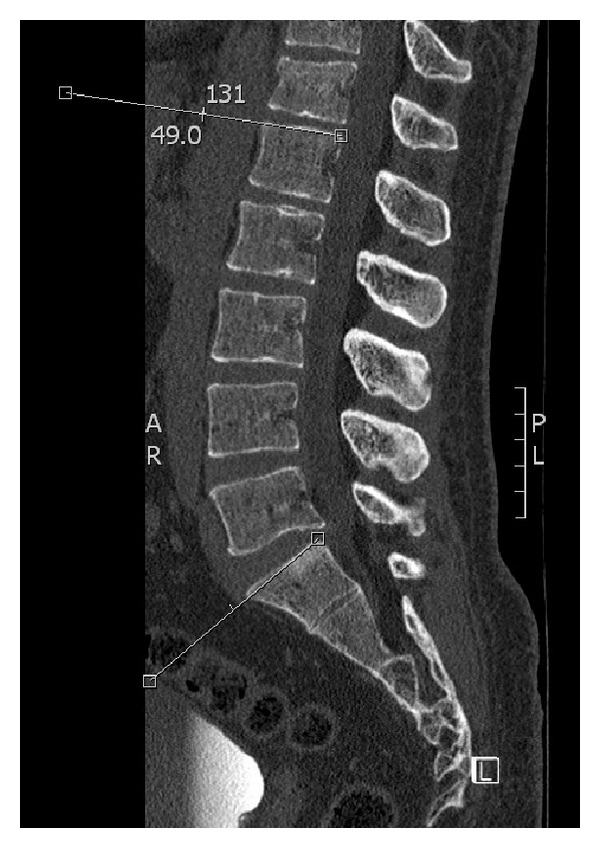
Anatomical Cobb angle measurement by CT.

**Figure 4:**
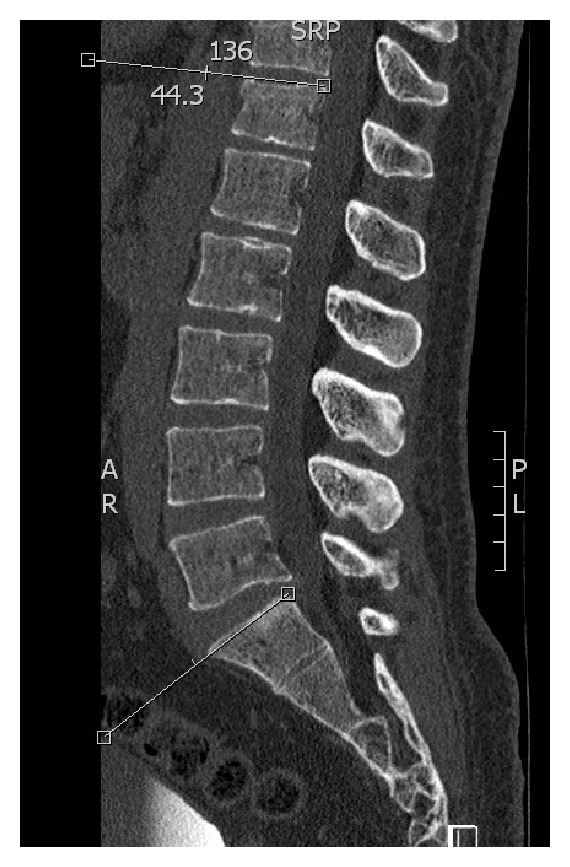
Functional Cobb angle measurement by CT.

Both the anatomical and functional Cobb angles were measured in the sagittal planes of the corresponding CT scans using the same PACS system. The angles were measured similarly to those measured by the conventional radiographs using the previously described landmarks. Thereby, the most central cut between the left and right vertebral edges was used for the measurements (Figures 3 and 4).

### 2.4. Statistical Analysis

All statistical analysis was performed by a professional biomedical statistician using SPSS (IBM SPSS Statistics for Windows, Version 21.0., Armonk, NY: IBM Corp.). Limits of agreement of ±3° and 5° between the conventional radiographs and CT scan measurements were defined, and intraindividual differences between conventional radiographs and CT measurements were calculated. All tests were performed at a significance level of *α* = 0.05. Confidence intervals were computed at a confidence level of 95%.

## 3. Results

In group A patients (with vertebral fractures), the mean number of fractures was 1.3 (1–6), and the most commonly fractured vertebrae were L1 (31.7%), L2 (30.7%), and L3 (14.9%). The mean anatomical and functional Cobb angles on conventional radiographs were 43.9° and 43.8°, respectively, whereas the mean angle on CT was 47.6° for both the anatomical and the functional measurements. The mean intraindividual difference ([°] conventional radiographs minus [°] CT) was −3.8° for the anatomical and the functional Cobb angles. Within a range of ±3° only 26 (anatomical Cobb angle) and 28 (functional Cobb angle) of the 101 patients in the fracture group were within these limits of agreement. Withregard to a threshold set at ±5°, 46 (anatomical Cobb angle) and 47 (functional Cobb angle) of the intraindividual differences were within the tolerable range.

In group B patients (without fractures), the mean anatomical and functional Cobb angles on the conventional radiographs were 48.0° and 48.2°, respectively. On CT scans, the mean anatomical and functional angles were 50.1° and 49.6°, respectively. The mean intraindividual difference was −2.1° for the anatomical and –1.5° for the functional Cobb angle. With regard to the anatomical Cobb angle, only 14 (26.9%) of the 52 patients in this group showed an intraindividual difference within the ±3° margin, whereas measurements of the functional Cobb angle yielded only 13 (25%) data sets that were within the same range. At a threshold of ±5°, 25 (48%) patients with functional and anatomical Cobb measurements were observed within the limits of agreement, and 27 patients (52%) were outliers.

**Figure 5:**
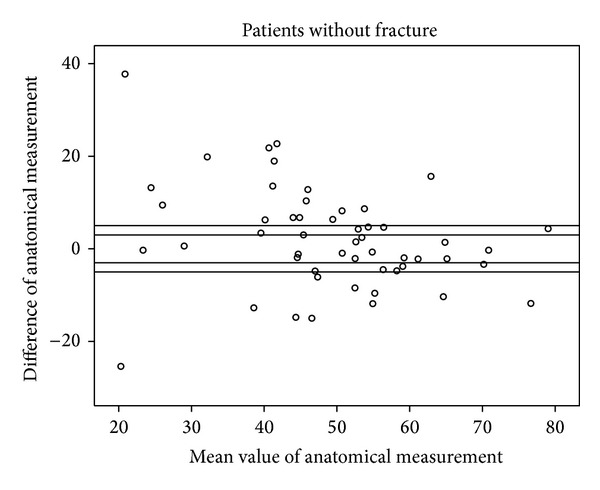
Scattergram of anatomical measurements in patients without fracture with acceptable margins of ±3° and ±5°.

**Figure 6:**
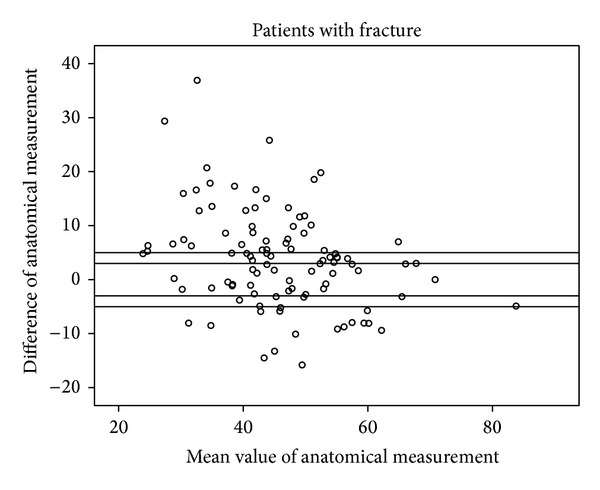
Scattergram of anatomical measurements in patients with fracture with acceptable margins of ±3° and ±5°.

**Figure 7:**
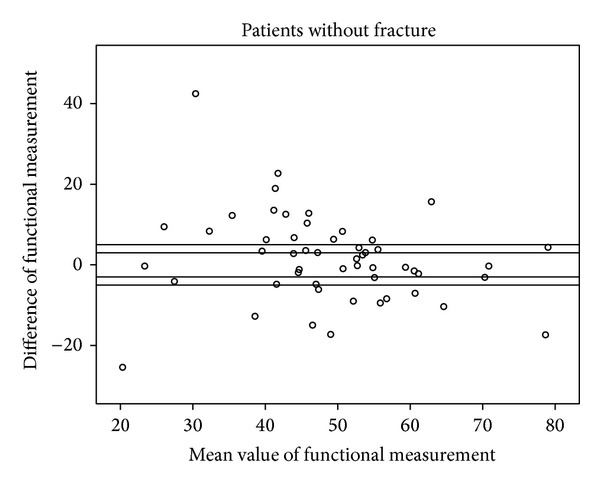
Scattergram of functional measurements in patients without fracture with acceptable margins of ±3° and ±5°.

**Figure 8:**
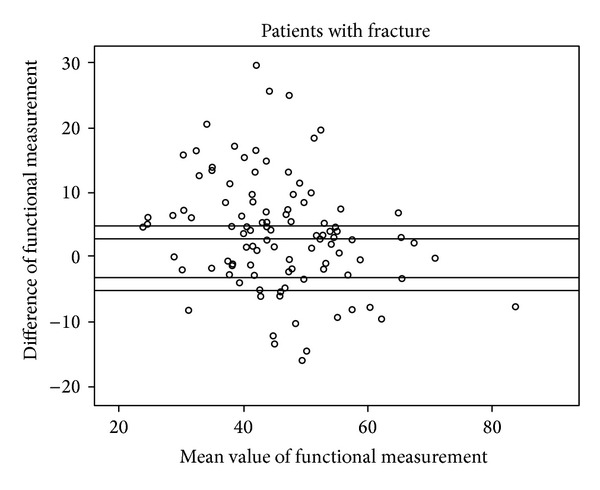
Scattergram of functional measurements in patients with fracture with acceptable margins of ±3° and ±5°.

Figures 5, 6, 7, and 8 show the distinct heterogeneity of measurements with respect to the levels of agreement.

## 4. Discussion

The conservative treatment of vertebral fractures requires sequential radiographic followup to evaluate the potential deterioration of posttraumatic lordosis and loss of vertebral height [10–13]. Changes to the individual treatment protocols and possible secondary surgical intervention often rely on the accurate assessment of the segmental and/or overall Cobb angle. In routine clinical practice, an increase of more than 2-3° in the Cobb angle that occurs within a follow-up period is usually considered to be significant and thus this measurement may exceed the accuracy of technical imaging. Cobb angle measurement errors in the assessment of scoliosis have been reported to be between 2° and 4° [14, 15]. The scarce available data comparing upright sagittal radiographs with supine measurements showed a difference of 3.1° less thoracic lordosis in the supine images of asymptomatic volunteers compared to images obtained in the upright position. With a decrease of the Cobb angle of –5.5° in the supine lumbar spine, the difference was even more pronounced. Although the differences were obvious, the authors of this study concluded that it might be possible “to use the supine radiographs to measure and plan treatment for the upright sagittal thoracolumbar spine” [2]. Another study compared not only the change in the sagittal geometry from the supine to the upright position but also the different radiological modalities using supine MRI and upright conventional radiographs. An overall difference between the angles of the lumbar lordosis in the standing and the supine position with fully extended lower extremities was found to be 3 degrees [3].

**Figure 9:**
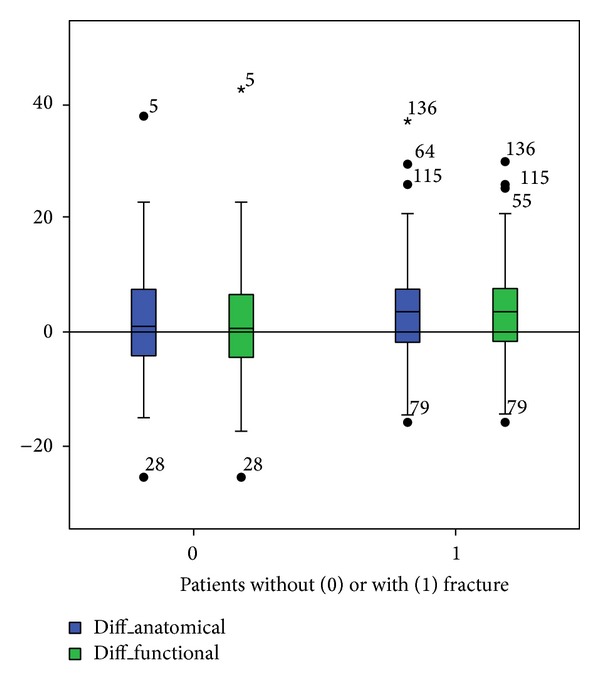
Differences in the Cobb angle measurements between the study groups.

In our study, we considered a range of 3° to be acceptable for both the anatomical and functional Cobb angles between the upright conventional sagittal radiographs and the supine CT measurements (Figure 9). To analyze the effect of an increased limit of agreement, we implemented a cut-off level of ±5° as well.

Using the two different cut-off levels, our results demonstrated that the mean differences of −3.8° in the fracture group (group A) and −2.1° and −1.5° in group B did not significantly exceed the acceptable range of ±3°. However, when all of the measurements were considered using either the functional or anatomical Cobb angle, only about one quarter of the intraindividual values remained within the ±3° range, and only about half of the measurements were within the ±5° limit of agreement.

The remaining individuals had significant divergences up to 42.5° between conventional radiographs and CT. Although the mean values are nearly (group A) or within (group B) the acceptable limit of agreement, the extensive heterogeneity of measurements obtained from the different body positions and radiological modalities in the vast majority of our study population cast doubts on the usefulness of WBCT as a baseline radiological assessment for vertebral fractures when follow-up imaging consists of upright sagittal conventional radiographs. This extensive heterogeneity of measurements has an important impact on clinical practice. There may be an increased risk of missing a clinically relevant vertebral body collapse that would have benefited from surgical intervention by obtaining an upright X-ray at followup and comparison with a previously obtained supine WBCT [13].

The limitations of the present study are the retrospective design and, further, the inability to distinguish between the effects of body position within one radiological modality or between the modalities using the same body position. Additionally, most of the fractures in our study population occurred in the lumbar spine; therefore, applicability to the thoracic spine may be limited. Of note, the Cobb angle has a key role in measurement of vertebral body collapses, which, in turn, influences sagittal spine lordosis. Even though disc degeneration also affects Cobb angles and sagittal spine lordosis, it may be assumed that disc degeneration in our patients remained steady between acquisition of the WBCT and X-ray. However, we were not able to compare disc heights and degeneration in supine WBCT and upright X-ray. Therefore, the impact of disc degeneration on the comparison of supine and upright radiographs remains unknown and further studies are recommended.

## 5. Conclusion

The use of supine CT measurements as a baseline assessment of the sagittal lordosis of the injured thoracolumbar spine does not appear to be appropriate when conventional upright sagittal plane radiographs are used for follow-up measurements. Obtaining additional standing conventional radiographs before the discharge of hospitalized patients should therefore be considered as routine clinical practice in the conservative treatment of vertebral fractures.

## References

[1] Venugopal K, Kinghorn AF, Emordi CE, Atkinson PR, Kendall RJ (2012). An evaluation of the use of whole-body computed tomography in trauma patients at a United Kingdom trauma centre. *European Journal of Emergency Medicine*.

[2] Wood KB, Kos P, Schendel M, Persson K (1996). Effect of patient position on the sagittal-plane profile of the thoracolumbar spine. *Journal of Spinal Disorders & Techniques*.

[3] Andreasen ML, Langhoff L, Jensen TS, Albert HB (2007). Reproduction of the lumbar lordosis: a comparison of standing radiographs versus supine magnetic resonance imaging obtained with straightened lower extremities. *Journal of Manipulative and Physiological Therapeutics*.

[4] Peterson MD, Nelson LM, McManus AC, Jackson RP (1995). The effect of operative position on lumbar lordosis: a radiographic study of patients under anesthesia in the prone and 90-90 positions. *Spine*.

[5] Mauch F, Jung C, Huth J, Bauer G (2010). Changes in the lumbar spine of athletes from supine to the true-standing position in magnetic resonance imaging. *Spine*.

[6] Eun SS, Lee HY, Lee SH, Kim KH, Liu WC (2012). MRI versus CT for the diagnosis of lumbar spinal stenosis. *Journal of Neuroradiology*.

[7] Magerl F, Aebi M, Gertzbein SD, Harms J, Nazarian S (1994). A comprehensive classification of thoracic and lumbar injuries. *European Spine Journal*.

[8] Heinzelmann M, Wanner GA (2008). *Thoracolumbar Spinal Injuries*.

[9] Roussouly P, Gollogly S, Berthonnaud E, Dimnet J (2005). Classification of the normal variation in the sagittal alignment of the human lumbar spine and pelvis in the standing position. *Spine*.

[10] Resch H, Rabl M, Klampfer H, Ritter E, Povacz P (2000). Surgical vs. conservative treatment of fractures of the thoracolumbar transition. *Unfallchirurg*.

[11] Farcy J-PC, Weidenbaum M, Glassman SD (1990). Sagittal index in management of thoracolumbar burst fractures. *Spine*.

[12] Sutherland CJ, Miller F, Wang GJ (1983). Early progressive lordosis following compression fractures. Two case reports from a series of ’stable’ thoracolumbar compression fractures. *Clinical Orthopaedics and Related Research*.

[13] Al-Khalifa FK, Adjei N, Yee AJ, Finkelstein JA (2005). Patterns of collapse in thoracolumbar burst fractures. *Journal of Spinal Disorders and Techniques*.

[14] Carman DL, Browne RH, Birch JG (1990). Measurement of scoliosis and lordosis radiographs. Intraobserver and interobserver variation. *Journal of Bone and Joint Surgery (American)*.

[15] Morrissy RT, Goldsmith GS, Hall EC, Kehl D, Cowie GH (1990). Measurement of the Cobb angle on radiographs of patients who have scoliosis. Evaluation of intrinsic error. *Journal of Bone and Joint Surgery (American)*.

